# Nutritional composition of substitutes for meats and sausages on the German market

**DOI:** 10.1093/eurpub/ckac131.236

**Published:** 2022-10-25

**Authors:** C Gréa, S Roser, I Hoffmann, S Storcksdieck genannt Bonsmann

**Affiliations:** 1 Nutritional Behaviour, Max Rubner-Institut, Karlsruhe, Germany; 2 Presidential Office, Max Rubner-Institut, Karlsruhe, Germany

## Abstract

**Background:**

Substitutes for meats and sausages are growing both in demand and supply in Germany. Monitoring their nutritional composition helps characterise this novel product group in terms of its dietary contribution.

**Methods:**

Data on substitutes for meats and sausages were collected in a baseline survey in 2016 and an in-depth follow-up survey in 2021. In both surveys, mandatory nutrition declaration (“Big 7”) and other packaging information were collected via online research and supermarket visits. Products were categorised according to the substituted animal products and their contents of energy, fat, saturated fatty acids, and salt investigated. Changes in energy and nutrient contents between baseline and follow-up survey were assessed statistically using Welch’s t-test.

**Results:**

The follow-up survey included 421 meat substitutes and 292 sausage substitutes, split into 27 subgroups. Substitutes for meat products like meat strips and schnitzel show widely varying energy contents. Sausage substitutes show higher medians for fat, saturated fatty acids and salt than meat substitutes; spans of salt content (0,1-4,0 g per 100 g product) and saturated fatty acids (0,1 g - 23,0 g per 100 g product) are particularly wide. Relative to the baseline survey, which included 69 meat substitutes and 61 sausage substitutes, the follow-up revealed significantly higher contents of energy and saturated fatty acids overall and in some subgroups (e.g. nuggets and burger patties). The sole significant reduction was seen for energy in substitutes of precooked sausages.

**Conclusions:**

The observed wide spans of energy and nutrient content imply 1) the potential to reformulate substitutes for meats and sausages at the top end of the spectrum 2) the availability of healthier choices within the various subgroups. The increases shown in energy and saturated fatty acids content warrant further monitoring.

**Key messages:**

• Wide spans of energy and nutrient contents reveal the potential for the development of more nutritionally favourable substitutes for meats and sausages.

• As substitutes for meats and sausages are perceived to have nutritional advantages the observed increases in energy and saturated fatty acids contents over time should be further monitored.

